# Analysis of the Strength and Quality Properties of Welded PVC Profiles with Glass Fiber Composite Reinforcement in the Context of Milling and Weld Head Feed

**DOI:** 10.3390/ma18061297

**Published:** 2025-03-15

**Authors:** Marek Kozielczyk, Kinga Mencel, Jakub Kowalczyk, Marta Paczkowska

**Affiliations:** 1 Faculty of Civil and Transport Engineering, Institute of Machines and Motor Vehicles, Poznan University of Technology, 60-965 Poznan, Poland; jakub.kowalczyk@put.poznan.pl (J.K.); marta.paczkowska@put.poznan.pl (M.P.); 2 Faculty of Mechanical Engineering, Institute of Material Technology, Poznan University of Technology, 60-965 Poznan, Poland; kinga.mencel@put.poznan.pl

**Keywords:** polyvinyl chloride, window profile, weld, breaking load, strength, welding head speed

## Abstract

Building materials, including polyvinyl chloride (PVC), play a key role in construction engineering, influencing the durability, esthetics, and functionality of structures. PVC stands out for its lightness, thermal insulation, and corrosion resistance. This makes it competitive with wood, aluminum, or steel, particularly in the manufacture of window joinery. One of the key technological processes in the processing of PVC profiles is welding, the quality of which depends on the precise control of parameters such as the temperature, time, and pressure regulating the speed of the welding heads. In modern welding machines, the use of servo drives guarantees the adequate precision and repeatability of the process, which allows better adjustment to technological requirements than in older machines. This study aimed to determine the effect of the heating head feed rate for selected milling depths on the quality and strength of window frame welds. A criterion in the assessment of the strength of the window frames was the result of failure load tests on the welds. In addition, the tests took into account the quality of the welds. The tests showed that the welding head feed rate of 0.25 mm/s generated the highest-quality welds, taking into account the continuity and symmetry of the weld and its highest failure load. When milling the composite to a depth of 1 mm, the average value of the failure load was 3637 N. Meanwhile, for speeds of 0.19 mm/s and 0.31 mm/s, it was 3157 N and 3033 N, respectively. For the 0.5 mm milling variant and without milling the composite, the average load values were significantly smaller.

## 1. Introduction

Materials play a key role in structural engineering, determining the durability, functionality, and esthetics of structures. It is through these that structures and products obtain their basic properties, such as strength, resistance to atmospheric conditions, or thermal insulation [1]. Materials influence their ability to carry loads, which is crucial in terms of safety [1,2]. When properly selected, they form not only the basis of a structure but also an element of visual space design. The wide range of materials currently available means that the technical, economic, and esthetic requirements can be fully met [3]. An important aspect is their resistance to wear and tear, which guarantees the longevity of the building [2,3]. Furthermore, modern materials increasingly also have ecological properties, which is part of the trend towards sustainable construction [4,5,6,7]. This is why choosing the appropriate materials is fundamental to any construction project.

One of the key construction materials is polyvinyl chloride, more commonly known as PVC [8,9,10]. This widely used material is applied, among others, in the manufacture of window profiles [11], where its unique properties ensure high functionality [12]. PVC is distinguished by its low density, which translates into its relatively low component weight compared to those produced from other materials, such as aluminum or steel; its corrosion resistance; its excellent thermal insulation; and its ease of processing [13]. These features make it competitive with materials such as wood, aluminum, and steel. Compared to many of these, it is easier to process and more resistant to weather conditions [13,14]. PVC production has been growing rapidly in recent years, driven by the increasing demand for energy-efficient and durable solutions [15,16]. In addition to its favorable price, this material is also gaining recognition due to its recyclability, making it a future-proof solution in the context of sustainable development [17,18,19]. Therefore, PVC shows innovation potential, particularly in identifying alternative solutions.

The authors of [20] discussed welding methods for plastics, with a particular focus on PVC. They focused on techniques such as hot gas, friction, and hot plate welding and key parameters such as the welding temperature and speed. It was emphasized that only thermoplastics are suitable for welding, while thermosetting plastics can only be joined mechanically or by an adhesive. The need for further research into the influence of the process parameters on the joint quality was pointed out, given the increasing role of plastics in industry. Hot air welding was found to be the most cost-effective and reliable method for PVC. However, it must be taken into account that such a method cannot be used under all conditions. The production conditions for PVC windows are most often characterized by the welding of profiles with four heads forming four corners at the same time. Thus, the use of a hot air nozzle is very limited under these conditions. In addition, it is not entirely economically justifiable.

The authors of [21] investigated the effect of low winter temperatures on the thermal deformation of PVC window profiles and developed an engineering methodology for their calculation under temperature loading. The case of a steel-core window mullion under thermal bending was analyzed using two calculation approaches: numerical–analytical and simplified analytical. To verify the methodology, temperature load tests were carried out on a double-hung window. The calculation and test results showed a slight discrepancy: 10.6% for the numerical–analytical method and 16.2% for the simplified method. The tests confirmed the assumption that the thermal deformation of the mullion could be calculated without taking into account the stiffness of the adjacent sashes, as both the sashes and the mullion deformed uniformly under the influence of the temperature, without transmitting mechanical forces to each other. The results show the need to take thermal deformation into account in the design of PVC windows, which has not been the case until now. Thanks to this new methodology, it is possible to predict deformations more accurately and improve the functionality of windows under severe climatic conditions. This research took into account a window profile reinforced with a steel profile. However, the behavior of a profile reinforced with a composite reinforcement was not investigated.

The authors of [22] investigated the use of closed steel reinforcement profiles in PVC window construction, comparing them with open profiles in terms of stiffness and torsional and bending strength. PVC profiles, due to their low Young’s modulus, have low stiffness and strength, leading to deformation during installation, especially under high wind loads. Steel reinforcements are the standard solution, but the currently used open profiles have low torsional stiffness, which limits their effectiveness. Studies have shown that closed profiles exhibit torsional stiffness that is tens or even hundreds of times higher compared to open profiles of the same size and wall thickness. Closed profiles have significantly lower stress and significantly higher bending and torsional stiffness. However, closed profiles have non-linear stiffness characteristics, and, beyond a torsion angle of about 10°, their ability to transmit torsional moments decreases. Despite the higher costs and technological problems, the use of closed steel or glass fiber-reinforced composite profiles is particularly justified in the construction of large windows subjected to high wind loads.

Another study carried out in [23] dealt with the thermal deformation of PVC window profiles reinforced with a metal core, particularly in the context of the action of negative external temperatures. A methodology was presented for the analytical calculation of the deformation, taking into account the nature of the forces transmitted by the PVC profile to the core during thermal bending. The highest forces were found at the extreme attachments, which had a decisive influence on the deformation. Mathematical models were developed to calculate the longitudinal forces resulting from the differences in the thermal shrinkage of the PVC and the core and thermal bending of the profile. To increase the accuracy of the calculations for long profiles, a physical model of the joint mechanical action of the PVC profile and the core was proposed. A comparison of the results obtained from the proposed method’s calculations and 3D modeling showed agreement, with an error of less than 10%. This methodology will allow earlier design decisions, reducing the need for costly laboratory tests and supporting the development of modern window designs that take thermal loads into account.

It can therefore be concluded that both the temperature and heat transfer play a key role in the formation of the profile during operations [23]. The process of welding PVC profiles with composite inserts is challenging due to the different melting temperatures of the two materials. The melting point of PVC is significantly lower than that required to melt the composite. This can cause difficulties during welding [24], and it is challenging to achieve a direct increase in temperature without the risk of depolymerizing the PVC. Therefore, to optimize the joining process, a milling method for the composite insert is proposed. A key aspect of this method is the precise selection of parameters such as the speed of the welding heads and the milling depth. Heat transfer plays a crucial role, as it influences the melting of the PVC profiles and allows correct bonding to the composite. It is necessary to study the phases of heat transfer, which are broken down into specific stages to better understand the mechanisms of the temperature’s effects on materials. The first step in this process is to analyze the destructive loads to understand the behavior of the material under welding conditions. The results of these studies can form the basis for further material analysis to confirm the effect of the head feed on the weld quality.

The articles presented so far on welding technology—or, more broadly, on PVC window technology—focus mainly on steel-reinforced profiles. They deal with mechanical strength, the influence of the temperature, thermal insulation, or the shape of the reinforcements included in the profile. The studies discussed do not consider constructional solutions for a profile reinforced with a composite insert.

This solution makes it possible to replace the steel profile with a composite reinforcement, but the welding procedure generates an additional problem [24]. This is an issue concerning the two different materials, i.e., the PVC profile and the glass fiber-reinforced composite, subjected to the same welding procedure. The different properties of these materials do not allow for a single preset melting temperature. The authors of [25] address this issue. Research has shown that the depth of treatment of composite inserts significantly affects the quality of the welded joint in PVC window frames. However, the mentioned research did not determine the optimal depth of milling. To do this, studies must be carried out that take into account the variation in another important welding process parameter: the speed of the welding heads.

Thus, this research aimed to determine the effect of the heating head feed rate for selected milling depths on the quality and strength of window frame welds.

## 2. Materials and Methods

### 2.1. Research Plan

**Figure 1:**
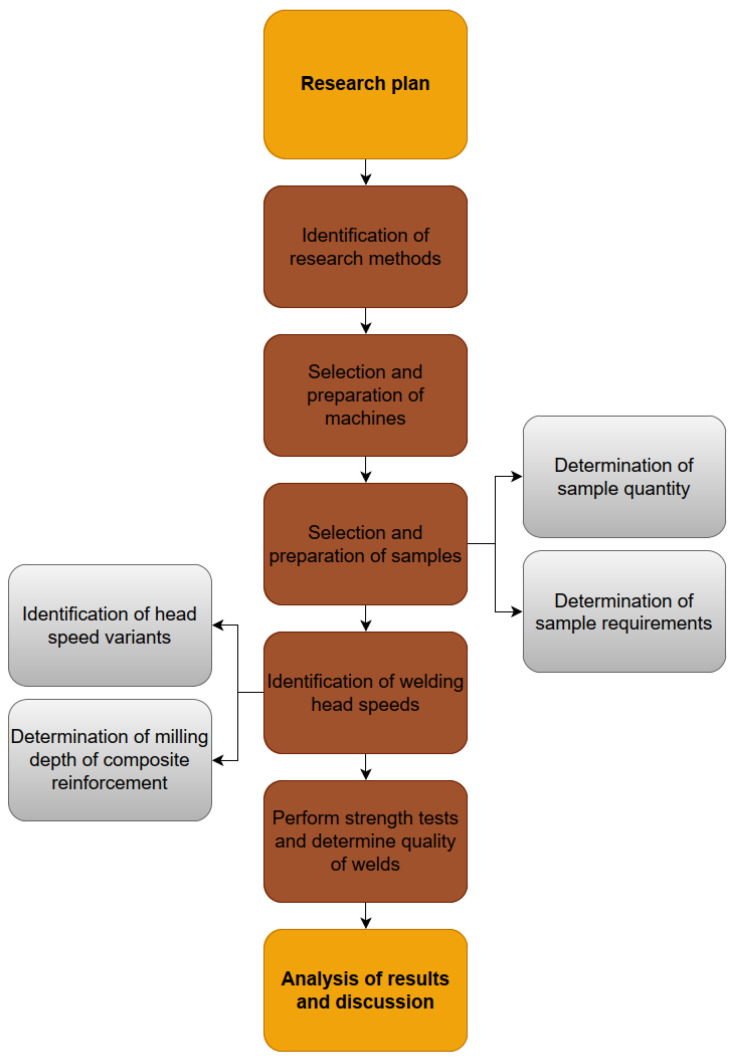
Research plan.

This research was carried out according to the scheme presented in Figure 1. The main research objectives included the selection of specimens (window profiles), the determination of the feed rate of the welding heads, the investigation of selected variants of the milling depth of the composite in the profile, and strength tests (bending by compression) on the window profiles of weld samples.

The tests involved the use of PVC window profiles with a composite insert. The research involved testing the feed rate of the welding heads in two different variants. The first variant assumed a higher head feed rate than the 0.25 mm/s initially adopted and the second considered a lower one. It was envisaged that each speed variant would be tested in the context of different milling depths of the composite. An examination of the above variants and a comparison of the results obtained, including those relating to tests of the head feed rate regarding the value adopted at the beginning of the study [25], should provide an answer to the question of which values of the head feed rate and depth of milling of the composite allow the highest values of the failure loads on the welds to be obtained.

### 2.2. Materials and Test Benches

The window profile of the frame was obtained through coextrusion technology with the glass fiber composite reinforcement “Powerdur Inside” from Aluplast GmbH (Karlsruhe, Germany). Its 85 mm depth, as well as its 70 mm width, allowed the structure to form 6 chambers inside the section. The profile was composed of unplasticized PVC. The material properties included a Vicat softening point of 80–84 °C, a notched impact of >40 kJ/m, and a flexural/tensile modulus of 2800 N/mm. The stability time at 200 °C was 40 ± 6 min, and the linear expansion coefficient in the temperature range of −30 °C to 50 °C was 7 × 10^−5^ 1/K [26].

**Figure 2:**
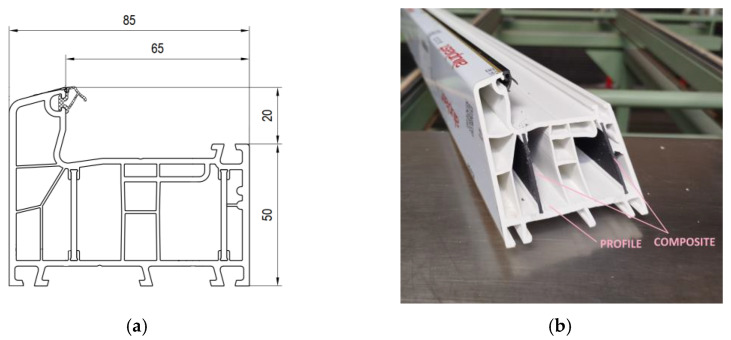
Drawing of the profile with composite inserts (**a**) and photo of the profile cross-section (**b**).

A drawing and photograph of the applied profile, including the area with composite inserts, are shown in Figure 2.

To maintain the appropriate static properties of buildings, PVC profiles are usually reinforced with steel profiles. Alternatively, these can be replaced with glass fiber-reinforced composite reinforcements, eliminating the need for additional reinforcements. The use of composite reinforcements brings numerous benefits, such as excellent thermal insulation properties, the removal of thermal bridges, and improved heat transfer coefficients by dispensing with steel. In addition, such a profile is significantly lighter than one containing traditional steel reinforcements.

A minimum of 3 window frames was included in all test variants, which corresponded to 12 individual weld samples, as required by the standard [27].

**Figure 3:**
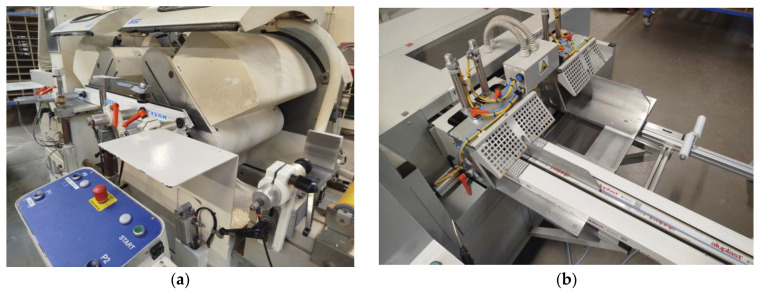
Profile machining: (**a**)—cutting; (**b**)—milling.

An automatic twin-head saw was used to cut the profiles (Figure 3a), namely the DS 150 Gamma from WEGOMA Polska (WEGOMA Weiss Fensterbau Maschinen GmbH, Bietigheim, Germany). The profiles were cut automatically, with the heads being calibrated before the process started. The machine allowed the required cutting dimension to several tenths of a millimeter. During cutting, the profile was stabilized by clamps in two planes: vertical and horizontal. The following cutting tool parameters were adopted: disc with carbide blades, disc diameter—550 mm, disc thickness—4.2 mm, number of teeth–120, tooth shape—flat trapezoidal, rotational speed of the cutting tool axle—3000 rpm, feed rate—45 mm/s.

An automatic two-spindle milling machine—namely the DFM-202/4 (URBAN Polska Sp. z o.o., Żary, Poland), shown in Figure 3b—was used to mill the composite reinforcing the profile. After positioning the profiles as shown, the composite was milled with carbide disc cutters. The machine allowed milling to be programmed to one decimal place. In the tests, an 80 mm disc milling cutter with a carbide blade with a 12 mm insert was used at a rotational speed of 18,000 rpm and a feed rate of 20 mm/s. The profiles in the machine were stabilized with pneumatic clamps in two planes.

**Figure 4:**
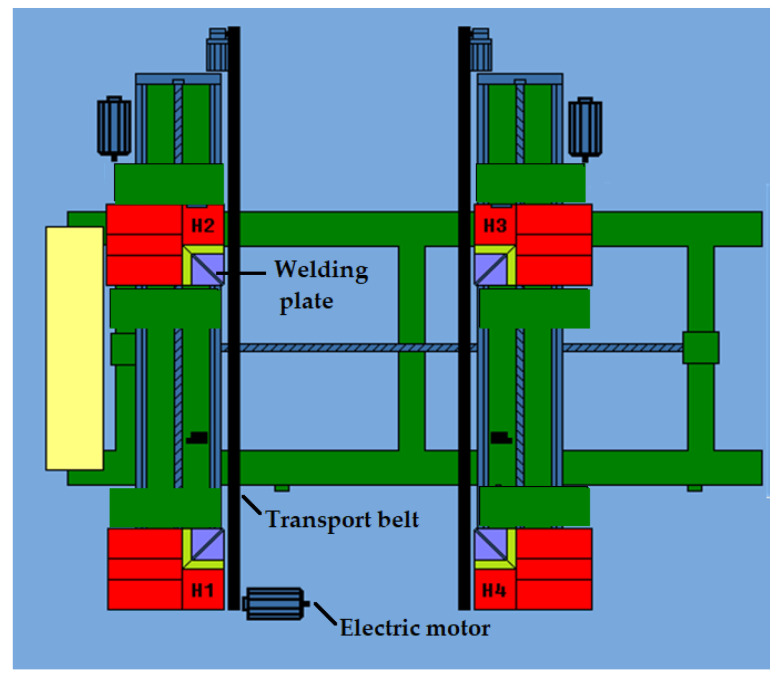
The 4-head welder model used in the tests. H1, H2, H3, H4—welding heads.

An automatic 4-head WSA 4RH/LH welding machine manufactured by WEGOMA Polska (WEGOMA Weiss Fensterbau Maschinen GmbH, Bietigheim, Germany) was used to carry out the welding cycle (Figure 4). All tests were carried out for a welding temperature of 264 °C and a melting time of 30 s. The feed rate of the welding heads was determined in two test variants. The values adopted are defined and described in the next subsection. Confidence intervals were used to determine the statistics, assuming a certain number of samples and a probability of 95%. To clean the welds after the welding process, an automatic corner cleaning machine, namely the WPCNC2/4 manufactured by WEGOMA Polska, was used.

**Figure 5:**
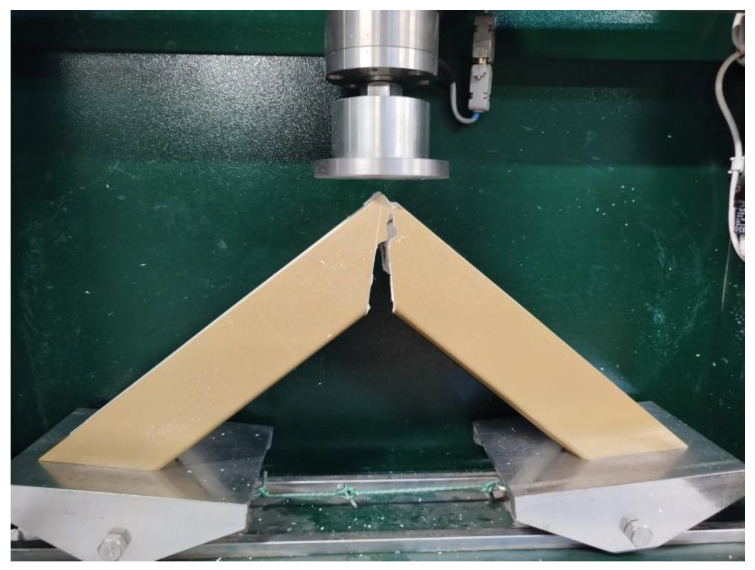
Example of a weld sample during a tensile bending test.

The strength tests (Figure 5) were conducted using the LN2000 corner breaker (PPHU DELTA, Ksawerów, Poland). The bases of the specimens were cut at an angle of 45° to the profile axis in such a way that their alignment was stable, without causing skewing. The breaking load was measured directly on the breaking punch, with a digital display showing the current value. The device also included a function to record the maximum value achieved during each test cycle. The compression bending testing machine had a load measurement range of 2 kN to 20 kN, with a test speed of 50 mm/min. The breaking load measurement accuracy was 10 N.

### 2.3. Methodology for Selection of Welding Head Speeds

The tested profiles met the assumed quality requirements, i.e., machinability at a minimum temperature of 18 °C [28]. This is the value assumed in the tests as being necessary for the correct processing of the profiles and operations such as cutting or welding, without the risk of technological defects. In addition, the profiles had the correct external dimensions, which was confirmed in preliminary tests. The profiles were rested and conditioned on a flat surface, which prevented bending, twisting, or deformation.

In the development of the methodology, the conclusions of a previously conducted study on the values of failure loads on welds, based on strength tests of sets of specimens that were subjected to different depths of milling of the composite reinforcement, were used [25]. In the study [25], it was shown that the highest average failure load values of between 3467 N and 3637 N were obtained for joints where the composite reinforcement profiles in each set of specimens were milled to a depth of 1 mm or 0.5 mm or without milling the composite. However, the tests did not indicate which of these could be considered the best. Therefore, the 0.5 mm step value of this parameter served as a starting point for the determination of the optimum speed of the welding heads. In this study, a weld head feed rate of 0.25 mm/s was initially assumed. Upon analyzing the available literature, no studies were encountered in which the effect of the head feed rate on the weld quality was assessed.

The significance of the head feed rate during welding on the quality of the welds was verified during the tests. A higher and lower value than that used by window manufacturers, i.e., 0.25 mm/s, was adopted. To estimate the value of this parameter, it was necessary to adopt values that would significantly change the value of the average failure load for the given sets of samples.

For the tests, the 3 mm allowance of the profile for a single weld element, which is commonly used in industry, was adopted (to form a classic weld, two elements with a 3 mm allowance on each side are needed). In the case of this parameter (3 mm allowance), two melting stages must be taken into account. The first is related to the melted profile on the heating mirror. The depth of this zone is 2.3 mm. This is the value at which the welding heads are actively involved, pushing the profile against the heating mirrors at a preset speed. This reflects the first phase of the set melting time, which lasts 12 s (this was a measured value). Once the 2.3 mm profile has been melted, the welding heads stop pressing the profile against the heating mirrors. A further melting stage follows to a depth of 0.7 mm, i.e., the remaining overmelt value. During the remaining melting time (18 s remaining of the assumed 30 s), the heat from the heating mirrors is transferred to the profile, causing it to soften. This study attempted to relate the 2.3 mm actual melting distance of the profile to the feed rate of the welding heads, with the knowledge that an important value for the milling depth of the composite, influencing the specific values of the failure loads, is at least 0.5 mm.

**Figure 6:**
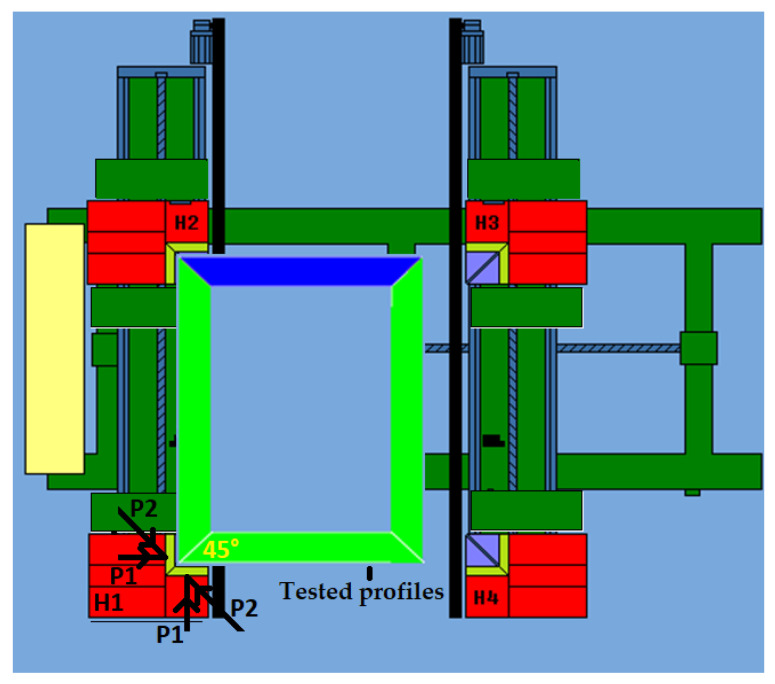
Schematic of the feed rate of the H1 welding head during the welding process: P1—physical feed rate, set and controlled; P2—feed rate resulting from the distribution of P1 and the angle of 45°; H1, H2, H3, H4—welding heads.

To achieve this, the pattern of forces acting on the profile was first determined (Figure 6). P1 is the physical feed rate (set and controlled). It is directed perpendicularly to the laid profile. The P1 output parameter, as recommended by the manufacturer, was 0.25 mm/s. P2 is the feed rate resulting from the distribution of the P1 force and the angle of 45°. This is the feed rate acting between the 45° cut surface of the profile and the heating mirror.

Due to the 45° cutting of the profile surface relative to its longitudinal axis, the position relative to the heating mirror, and the distribution of forces, the passive feed rate of the heads was parameter P2. On the other hand, the programmable parameter (active feed of the heads), from the operator’s point of view during the tests, was parameter P1. Thus, regarding the 2.3 mm melting distance covered by the profile, the passive feed rate parameter of the P2 heads was crucial. To determine the feed rate of the P2 heads, it was necessary to identify the relationship between the P1 parameter and the P2 parameter. The relationship between the parameters is shown in Equation (1).

(1) $$P2=P1\cdot {\cos45}^{{}^{\circ}}=0.25\frac{mm}{s}\cdot 0.71=0.18\frac{mm}{s}$$

To calculate the feed change of the welding heads, the 0.5 mm melting distance needed to be divided by the real profile’s melting time. The measured feed time of the welding heads was 12 s. However, it should be noted that the profile, relative to the heating mirror, is set at a certain distance for safety reasons when lowering the mirrors. The setting is defined as fixed in the standard process but can be changed in specific cases. The smaller the distance, the longer the real melting time. The distance measured with the gap gauge was 0.2 mm. Given that the P2 parameter was 0.18 mm/s, it had to be assumed that the first second of head movement was consumed by the profile reaching the heating mirrors. Therefore, the real melting time of the profile was 11 s. Consequently, the change in feed rate P2 of the welding heads can be determined by Formula (2).

(2) $$\Delta P2=0.5\;mm:11\;s=0.045\;\frac{mm}{s}$$

However, it should be taken into account that the possible parameter to be programmed into the machine was P1. To calculate the change in parameter P1, we use Formula (3).

(3) $$\Delta P1=\Delta P2:{\cos45}^{{}^{\circ}}=0.045\;\frac{mm}{s}:0.707=0.06\;\frac{mm}{s}$$

Accordingly, to melt approximately 0.5 mm of the profile at a constant head travel time of 12 s, the speed of the welding heads must be increased or decreased by at least 0.06 mm/s. Thus, the alternative speeds of the welding heads should be assumed to be 0.19 mm/s and 0.31 mm/s.

Accordingly, the options in the table below (Table 1) were adopted for this study.

## 3. Analysis of Results and Discussion

### 3.1. Test of Weld Fracture Loads for a Set Welding Head Speed of 0.31 mm/s When Milling the Composite Reinforcement to a Depth of 1 mm

The first case study investigated the failure loads of the welds at a given weld head feed rate of 0.31 mm/s. In addition, the composite reinforcement was milled to a depth of 1 mm. Table 2 shows the results in terms of the dimensional deviations for the length and width of the mold after welding relative to the nominal dimension in the individual machine axes.

The results in the table (Table 2) show that there is no difference greater than 1 mm between the set dimension and the final dimension after welding. This confirms that the form has been achieved within the dimensional tolerance of 1 mm.

**Figure 7:**
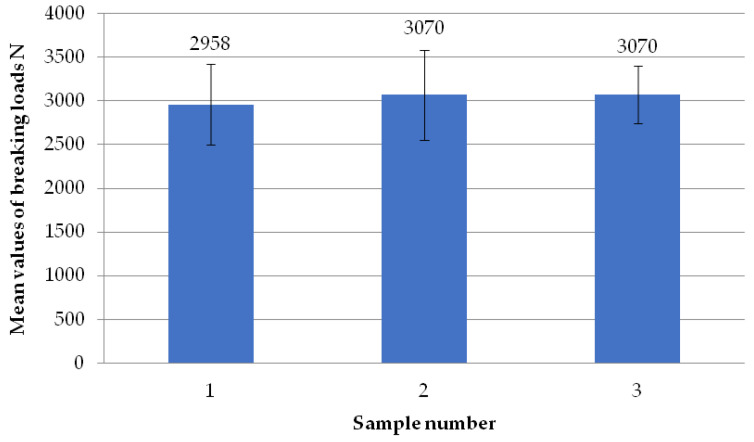
Average failure loads for sets of specimens at a set welding head speed of 0.31 mm/s with the milling of the composite reinforcement to a depth of 1 mm.

The figure below (Figure 7) shows the average failure loads on the sets of specimens for a set welding head speed of 0.31 mm/s when milling the glass fiber-reinforced composite to a depth of 1 mm. It can be seen that there are no significant differences in the values between the individual average loads. Two sets of specimens exceeded the breaking load threshold of 3000 N and one exceeded the threshold of 2950 N.

**Figure 8:**
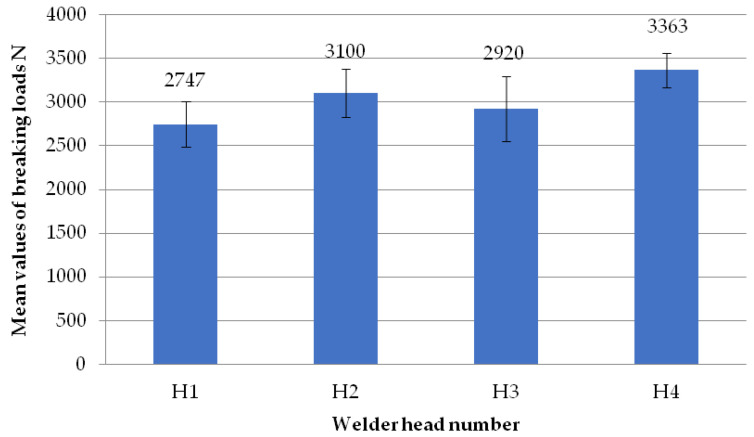
Average failure loads for sets of specimens at a set welding head speed of 0.31 mm/s with the milling of the composite reinforcement to a depth of 1 mm with welding head breakdown.

The figure below (Figure 8) shows the average failure loads on the sets of specimens for a set weld head speed of 0.31 mm/s when milling the glass fiber-reinforced composite to a depth of 1 mm per weld head. Heads H1, H2, and H3 achieved similar results. An analogous situation occurred for heads H2, H3, and H4. Only the results between heads H1 and H4 are significantly different. The reason for this could be the displacement of the profile on the spacer (defect in profile alignment) before the welding process, which is associated with the slight twisting of the profile. However, as shown in Table 1, the differences in the dimensional deviations do not exceed 1 mm, so this discrepancy can be ignored.

**Figure 9:**
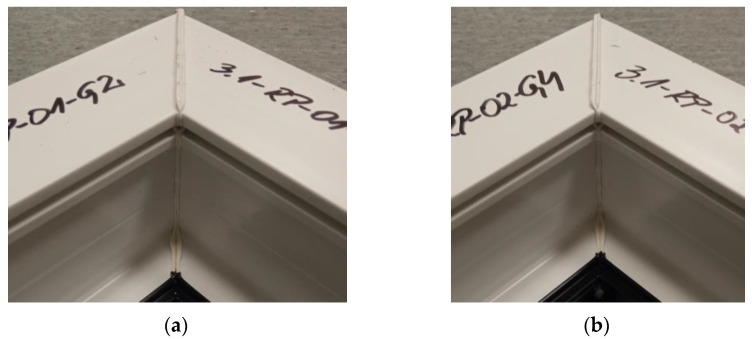
Two sample welds of reconnaissance test samples: (**a**)—for the second head, (**b**)—for the fourth head.

The figure below (Figure 9) shows two welds of two different specimens when the set speed of the welding heads is 0.31 mm/s and when milling the glass fiber-reinforced composite to a depth of 1 mm. The presence of a visible weld bead indicates material continuity affecting the failure load values of the welds. The white color of the weld bead indicates the correct choice of the temperature parameter. If the temperature of the welding process is too high, there is a risk of the depolymerization of the PVC profile. This manifests itself in the dark color of the welding seam.

### 3.2. Test of Weld Fracture Loads for a Set Welding Head Speed of 0.19 mm/s When Milling the Composite Reinforcement to a Depth of 1 mm

The second case considered was an investigation of the failure loads of the welds with a set feed rate of 0.19 mm/s for the welding heads. In addition, the composite reinforcement was milled to a depth of 1 mm. The table below (Table 3) shows the results in terms of the dimensional deviations for the length and width of the mold after welding relative to the nominal dimension in the individual machine axes.

Table 3 shows that the difference between the set dimension and the final dimension when the welding does not exceed 1 mm, confirming that the dimensional tolerance requirement of 1 mm is met.

**Figure 10:**
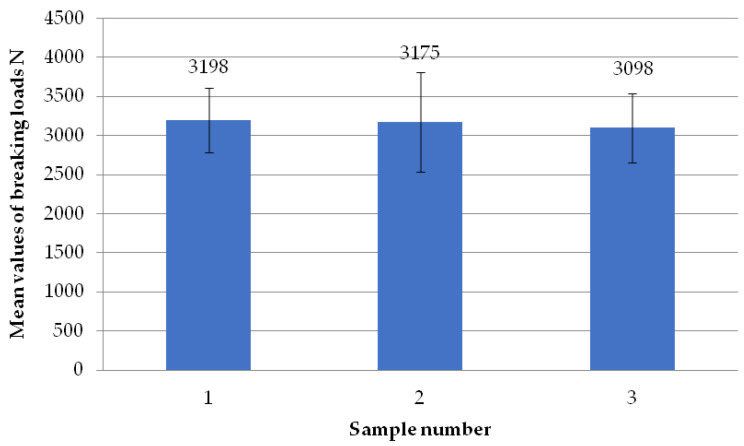
Average failure loads for sets of specimens at a set welding head speed of 0.19 mm/s with the milling of the composite reinforcement to a depth of 1 mm.

The figure below (Figure 10) shows the average failure loads on the sets of specimens for a set welding head speed of 0.19 mm/s when milling the glass fiber-reinforced composite to a depth of 1 mm. It can be seen that there are no significant differences in the values between the individual average loads. Two sets of specimens exceeded the failure load threshold of 3100 N, and two specimens exceeded the 3100 N threshold.

**Figure 11:**
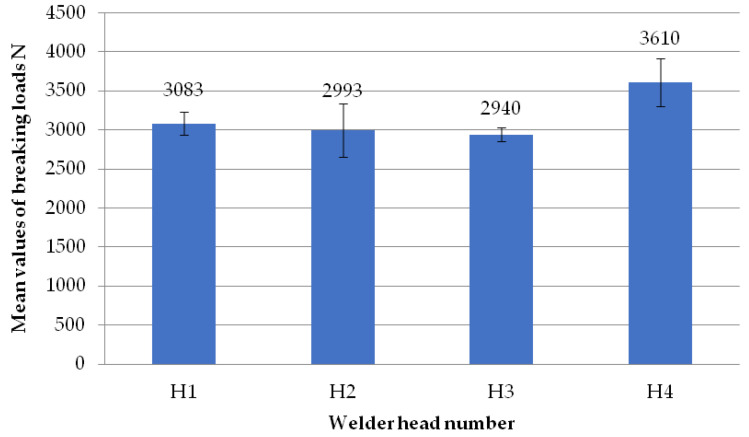
Average failure loads for sets of specimens at a set welding head speed of 0.19 mm/s with the milling of the composite reinforcement to a depth of 1 mm per weld head.

The figure below (Figure 11) shows the average failure loads on the sets of specimens for a set weld head speed of 0.19 mm/s when milling the glass fiber-reinforced composite to a depth of 1 mm per weld head. There is no significant difference between the average head loads H1, H2, and H3. All three recorded average results are above 2900 N, which is above our expectations. Head H4 has a higher average breaking load than the others. Its value is 3610 N. However, this value does not differ significantly from that of H2, which has an average value of 2993 N.

**Figure 12:**
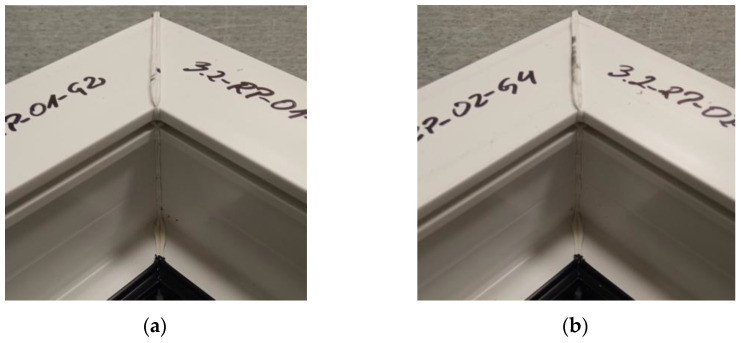
Two sample welds of reconnaissance test samples: (**a**)—for the second head, (**b**)—for the fourth head.

The figure below (Figure 12) shows two welds of two different specimens for a set welding head speed of 0.19 mm/s when milling the glass fiber-reinforced composite to a depth of 1 mm. The symmetry of the weld was investigated. As a result, it was confirmed that there was no displacement of the profiles during the welding process. The presence of a visible weld seam indicates material continuity, which affects the strength of the welds. In turn, the strength of the welds determines the value of the failure load. The white color of the weld bead indicates the correct choice of the temperature parameter.

### 3.3. Testing of Weld Failure Loads for a Set Welding Head Speed of 0.31 mm/s When Milling the Composite Reinforcement to a Depth of 0.5 mm

The third case considered was the investigation of the failure loads of the welds with a set feed of the welding heads of 0.31 mm/s. In addition, the composite reinforcement was milled to a depth of 0.5 mm. Table 4 shows the dimensional deviation results for the length and width of the mold after welding relative to the nominal dimension in each machine axis.

According to the data shown in the table (Table 4), the deviation between the set dimension and the one obtained after welding does not exceed 1 mm. This confirms that the dimensional tolerance of 1 mm is maintained.

**Figure 13:**
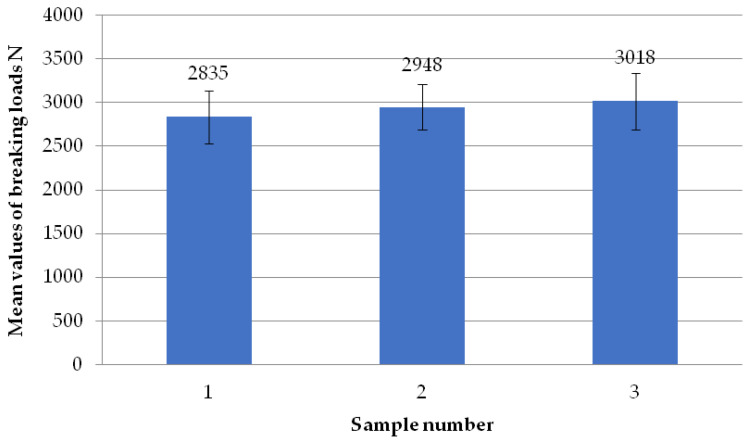
Average failure loads for sets of specimens at a set welding head speed of 0.31 mm/s with the milling of the composite reinforcement to a depth of 0.5 mm.

The figure below (Figure 13) shows the average failure loads of the specimen sets for a set welding head speed of 0.31 mm/s when milling the glass fiber-reinforced composite to a depth of 0.5 mm. It can be seen that there are no significant differences in the values between the individual average loads for all sets of samples. All sets exceeded the value of the average failure load of 2800 N. However, the third set of specimens exceeded the average breaking load value of 3000 N.

**Figure 14:**
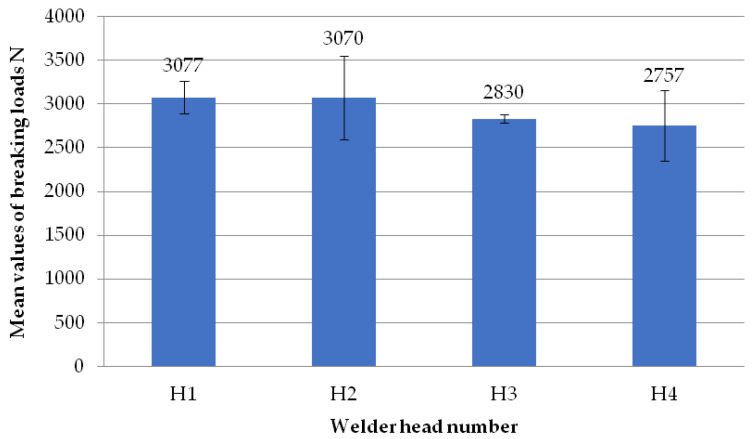
Average failure loads for sets of specimens at a set welding head speed of 0.31 mm/s with the milling of the composite reinforcement to a depth of 0.5 mm per weld head.

The figure below (Figure 14) shows the average failure loads of the sets of specimens for a set welding head speed of 0.31 mm/s when milling the glass fiber-reinforced composite to a depth of 0.5 mm per welding head. There are no significant differences between the average loads of all heads. The highest average value is found for the first head, at 3077 N. The lowest average load is found for the fourth head, at 2575 N. However, this difference is not significant concerning the results presented.

**Figure 15:**
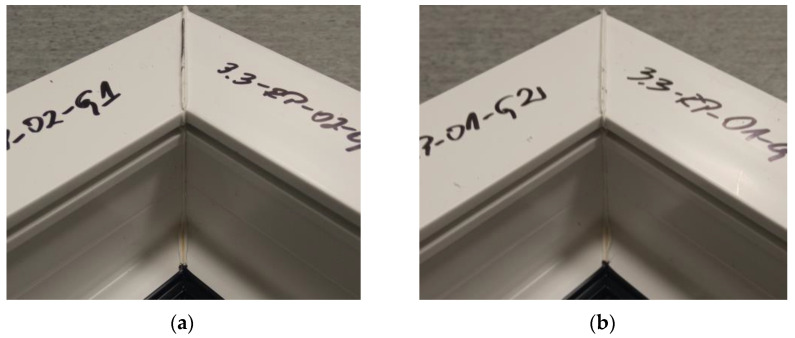
Two sample welds of reconnaissance test samples: (**a**)—for the first head, (**b**)—for the second head.

The figure below (Figure 15) shows two welds of two different specimens for a set welding head speed of 0.31 mm/s when milling the glass fiber-reinforced composite to a depth of 0.5 mm. As a result of testing the symmetry of the welds, confirmation of the correctness and symmetry of the weld was obtained. The material continuity was preserved due to the visible weld seam. Moreover, in this case, a white efflorescence could be noted, confirming the correctness of the adopted temperature parameter.

### 3.4. Testing of Weld Failure Loads for a Set Welding Head Speed of 0.19 mm/s When Milling the Composite Reinforcement to a Depth of 0.5 mm

The fourth case considered was the investigation of the failure loads on the welds with a set feed of the welding heads of 0.19 mm/s. In addition, the composite reinforcement was milled to a depth of 0.5 mm. Table 5 shows the results in terms of the dimensional deviations for the length and width of the mold after welding relative to the nominal dimension in each machine axis.

From the data presented in the table (Table 5), it can be seen that the difference between the set dimension and that obtained after the welding process does not exceed 1 mm. This proves that the required dimensional tolerance of 1 mm is maintained.

**Figure 16:**
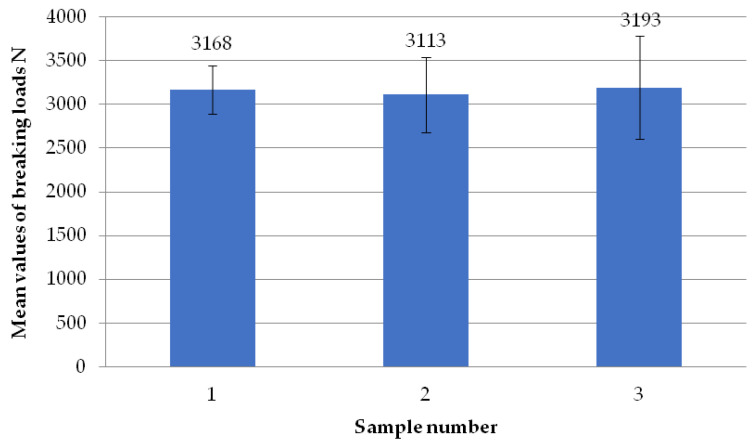
Average failure loads for sets of specimens at a set welding head speed of 0.19 mm/s with the milling of the composite reinforcement to a depth of 0.5 mm.

The figure below (Figure 16) shows the average failure loads of the sets of specimens for a set welding head speed of 0.19 mm/s when milling the glass fiber-reinforced composite to a depth of 0.5 mm. It is noted that the values of the average loads for all sets of samples are similar to each other and do not show significant differences. All three sets of specimens exceeded the average value of the breaking load of 3100 N.

**Figure 17:**
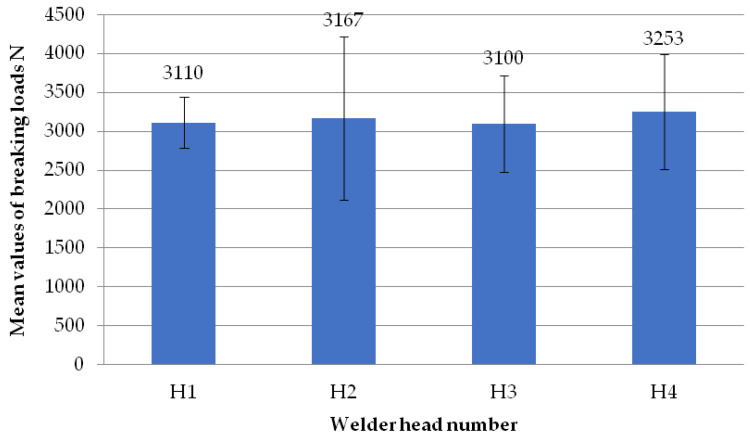
Average failure loads for sets of specimens at a set welding head speed of 0.19 mm/s with the milling of the composite reinforcement to a depth of 0.5 mm per weld head.

The figure below (Figure 17) shows the average failure loads on the sets of specimens for a set welding head speed of 0.19 mm/s when milling the glass fiber-reinforced composite to a depth of 0.5 mm per welding head. No significant differences could be found between the average loads of all heads. The highest average value was found for the fourth head, with a value of 3253 N. The next two heads, H1 and H3, obtained the same average loads of 3100 N. Head H2, on the other hand, obtained a value of 3167 N.

**Figure 18:**
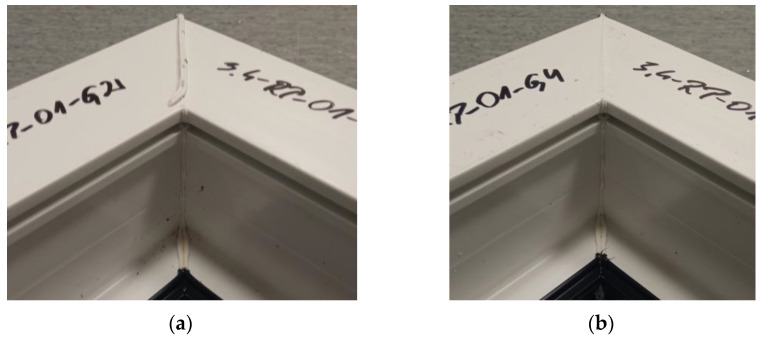
Two sample welds of reconnaissance test samples: (**a**)—for the second head, (**b**)—for the fourth head.

The figure below (Figure 18) shows two welds of two different specimens for a set welding head speed of 0.19 mm/s when milling the glass fiber-reinforced composite to a depth of 0.5 mm. An examination of the welds showed their correctness and symmetry. The continuity of the material was maintained, confirming the presence of a weld seam. In addition, a white discharge was noted, indicating the correct choice of the temperature parameter.

### 3.5. Testing of Weld Failure Loads for a Set Welding Head Speed of 0.31 mm/s Without Milling the Composite Reinforcement

The fifth case considered was a study of the failure loads of the welds with a set feed of the welding heads of 0.31 mm/s. The composite reinforcement was not milled. Table 6 shows the results in terms of the dimensional deviations for the length and width of the mold after welding relative to the nominal dimension in each machine axis.

Considering the data in the table (Table 6), it can be concluded that the difference between the set dimension and that obtained after the welding process does not exceed 1 mm. This confirms that the required dimensional tolerance of 1 mm is met.

**Figure 19:**
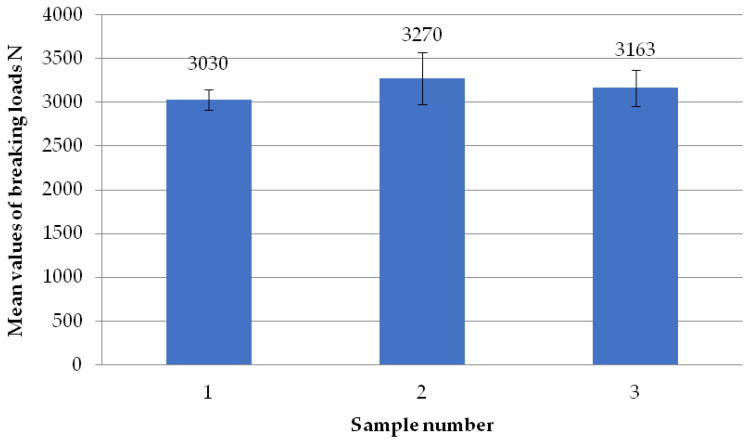
Average failure loads for sets of specimens at a set welding head speed of 0.31 mm/s without the milling of the composite reinforcement.

The figure below (Figure 19) shows the average failure loads of the sets of specimens for a set welding head speed of 0.31 mm/s without milling the glass fiber-reinforced composite. It is observed that the average load values in all sets of specimens are comparable and do not differ significantly. All three sets of specimens exceeded the average failure load value of 3000 N. In contrast, the other two sets reached an average value exceeding 3100 N.

**Figure 20:**
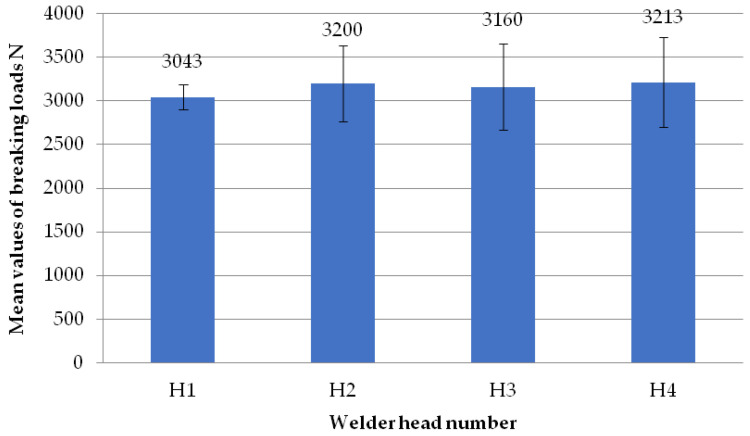
Average failure loads for sets of specimens at a set welding head speed of 0.31 mm/s without the milling of the composite reinforcement per weld head.

The figure below (Figure 20) shows the average failure loads of the sets of specimens for a preset welding head speed of 0.31 mm/s and without the milling of the glass fiber-reinforced composite, broken down into individual welding heads. All average loads represented by the individual welding heads had similar values. Each of them exceeded the 3000 N level. However, two of them, H2 and H4, reached the level of 3200 N.

**Figure 21:**
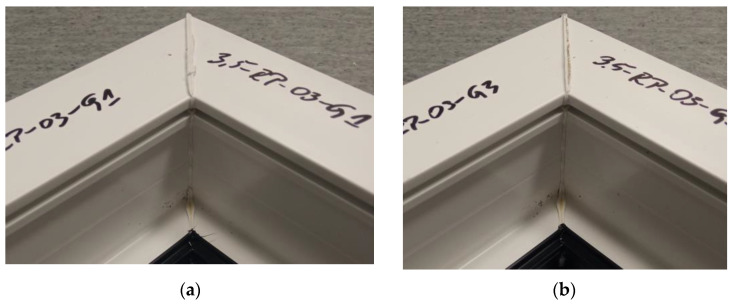
Two sample welds of reconnaissance test samples: (**a**)—for the first head, (**b**)—for the third head.

The figure below (Figure 21) shows two welds of two different specimens for a set welding head speed of 0.31 mm/s without milling the glass fiber-reinforced composite. An examination of the welds confirmed their correctness and symmetry. The preservation of material continuity was confirmed by the presence of a weld seam. In addition, a white efflorescence was observed, indicating the correct choice of the temperature parameter.

### 3.6. Testing of Weld Failure Loads for a Preset Welding Head Speed of 0.19 mm/s Without Milling the Composite Reinforcement

The sixth case considered was a study of the failure loads of the welds with a set feed of the welding heads of 0.19 mm/s. The composite reinforcement was not milled. Table 7 shows the results in terms of the dimensional deviations for the length and width of the mold after welding relative to the nominal dimension in each machine axis.

Analyzing the data in the table (Table 7), one can draw similar conclusions to those for the previous test variants. The results show that the difference between the set dimension and the one obtained after welding is within 1 mm, which indicates that the required dimensional tolerance of 1 mm is met.

**Figure 22:**
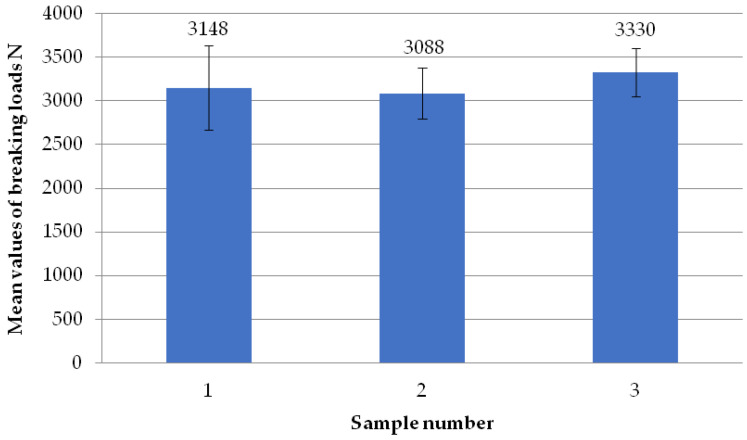
Average failure loads for sets of specimens at a set welding head speed of 0.19 mm/s without the milling of the composite reinforcement.

The figure below (Figure 22) shows the average failure loads of the sets of specimens for a set welding head speed of 0.19 mm/s and without milling the glass fiber-reinforced composite. Analyzing the results, it can be concluded that the average load values in all sets of samples were similar, with no noticeable differences. All sets obtained average loads exceeding 3000 N. One of them obtained an average value exceeding 3300 N.

**Figure 23:**
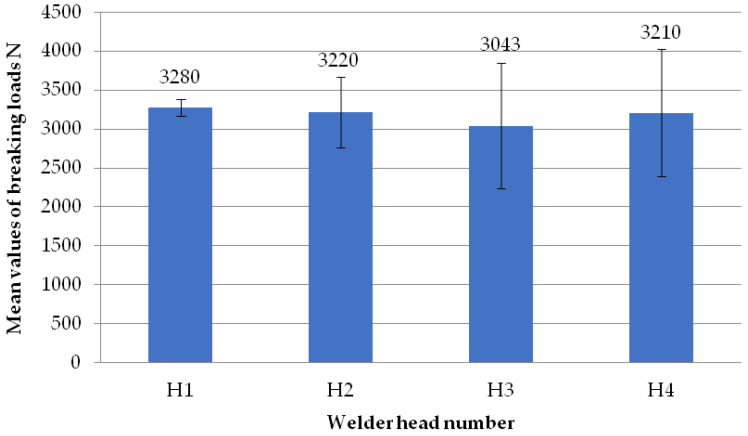
Average failure loads for sets of specimens at a set welding head speed of 0.19 mm/s without the milling of the composite reinforcement per weld head.

The figure below (Figure 23) shows the average failure loads of the sets of specimens for a preset welding head speed of 0.19 mm/s without milling the glass fiber-reinforced composite, according to individual welding heads. All heads exceeded the average failure load of 3000 N. The highest value was found for the H1 head, with a value of 3280 N. The lowest average value was found for the H3 head, with a value of 3043 N.

**Figure 24:**
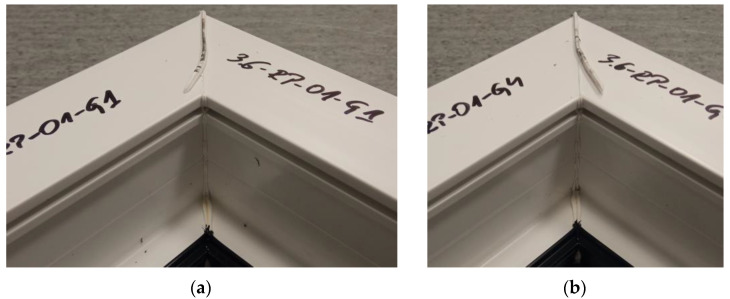
Two sample welds of reconnaissance test samples: (**a**)—for the first head, (**b**)—for the fourth head.

The figure below (Figure 24) shows two welds of two different specimens for a set welding head speed of 0.19 mm/s and without milling the glass fiber-reinforced composite. The tests carried out showed that the welds were appropriate constructed and symmetrical. The presence of the weld seams confirmed the material’s continuity, and the presence of a white discharge indicated the appropriately selected temperature parameter.

### 3.7. Summary Matrix of the Studies Conducted

**Figure 25:**
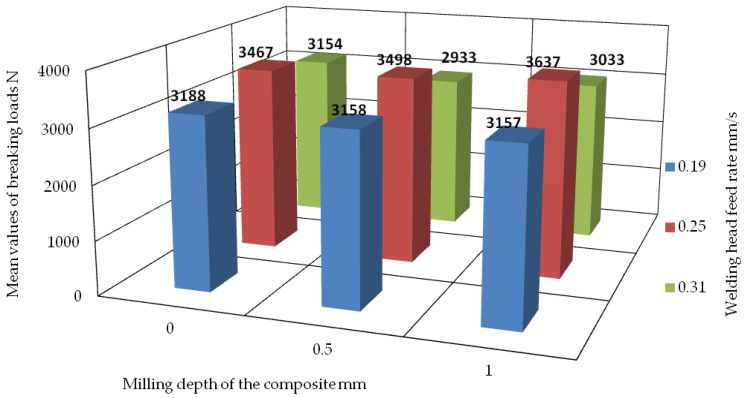
Summary of mean values of failure loads for sets of specimens in the context of the milling depth of the composite and the feed rate of the welding heads. The summary takes into account previously obtained results regarding failure loads [25] for a weld head feed rate of 0.25 mm/s.

The figure below (Figure 25) shows a summary of the average values of the failure loads of the welds, taking into account the depth of milling of the composite, i.e., 0.5 mm and 1 mm, and without the milling of the composite. We also show the speeds of the welding heads: 0.19 mm/s, 0.25 mm/s, and 0.31 mm/s.

Analyzing the test results for the composite milling depth of 1 mm, it can be concluded that there are differences in the values of the average failure loads for different welding head speeds. The lowest average values of the failure loads occurred at the feed values of 0.31 mm/s and 0.19 mm/s. In the first case, the average load value was 3033 N. However, for the speed of 0.19 mm/s, it was 3157 N. The highest average value of the failure load was found for the speed of 0.25 mm/s, and it was 3637 N. Thus, it can be concluded that the average value of the failure load for the speed of 0.25 mm/s is much higher than the others and significantly different from them.

From the figure (Figure 25), it is also possible to determine the average values of the failure loads on the welds under the milling of the composite reinforcement to a depth of 0.5 mm. Based on the analysis of the test results, it can be concluded that there are differences in the values of the average failure loads depending on the speed of the welding heads. Among the lowest, we note the average values of the failure loads recorded when the feed of the welding heads was 0.31 mm/s or 0.19 mm/s. These were analogous to the case in which the composite reinforcement was milled to a depth of 1 mm. In the first case, the average value of the load was 2933 N. However, for the speed of 0.19 mm/s, it was 3158 N. The highest average value of the failure load was for a speed of 0.25 mm/s, reaching 3498 N. However, this value was lower than the average failure load when milling the composite to a depth of 1 mm at the same speed as for the heads. It can be concluded that the average value of the failure load at a speed of 0.25 mm/s is much higher than for the other speeds and differs from them.

The figure (Figure 25) also presents the average values of the failure loads of the welds without the milling of the composite reinforcement. The results of the test analysis show that the average values of the failure loads vary depending on the speed of the welding heads. The lowest average values of the failure loads were once again recorded when the feed of the welding heads was 0.31 mm/s or 0.19 mm/s, as in the case of milling the composite reinforcement to a depth of 0.5 mm or 1 mm. In the first case, the average value of the load was 3154 N. However, for the speed of 0.19 mm/s, it was 3188 N. The highest average value of the failure load was for the speed of 0.25 mm/s and was 3467 N. This value was the lowest among all of the average failure loads for this head speed. However, the average value of the failure loads at 0.25 mm/s was significantly higher than for the other tested speeds. Moreover, it showed a significant difference in the comparison.

Analyzing the figure (Figure 25), it is clear that the optimal variant in terms of the feed rate of the welding heads is the value of 0.25 mm/s. Thus, it remains to evaluate the following variants: without milling, milling the composite to a depth of 0.5 mm, and milling the composite to a depth of 1 mm. Analyzing the results, it can be concluded that the lowest average value of the failure load is shown by the set of specimens without the milling of the composite. This value is 3467 N. The highest value was shown by the set of samples in which the composite was milled to a depth of 0.5 mm, and it amounted to 3498 N. The highest average value of the failure load was shown by the set of samples for which the composite was milled to a depth of 1 mm, and it was 3637 N. However, the differences in these values are so small that it is not possible to determine with certainty which variant yields the highest and most repeatable average value of the failure load.

Concerning the analysis presented, it can be concluded that the feed rate of the welding heads is one of the key parameters regarding the failure loads of heating welds. A head velocity value of 0.25 mm/s is appropriate for the adopted welding temperature of 264 °C and a profile melting time of 30 s due to the properties and their divergence between the materials, namely PVC and the glass fiber composite. The adopted temperature of 264 °C is appropriate, as confirmed by the visual inspection of the welds. However, it is insufficient to melt the composite reinforcing the profile. Such a high melting rate (0.31 mm/s) causes the composite to exhibit considerable resistance to the welding heads. Consequently, the reinforcement composite, which is a substitute for steel reinforcement, is unable to properly reinforce and form the weld. As a result, the value of the failure load on the welds decreases. On the other hand, a low melting speed (0.19 mm/s) results in unused heat energy coming from the hotplates. Consequently, the selected weld material is not sufficiently melted to form a fully durable weld.

## 4. Conclusions

Based on the study of the average values of the failure loads of welds when milling the composite reinforcement to a depth of 1 mm, it can be concluded that the average failure loads vary depending on the feed of the welding heads. For a feed rate of 0.25 mm/s, the highest values of the failure loads were obtained. Both a lower feed rate of 0.19 mm/s and a higher feed rate of 0.31 mm/s yielded lower-quality welds.

The results presented when milling the composite to a depth of 0.5 mm indicate the presence of differences in the average load values depending on the speed of the welding heads. For a head feed rate of 0.25 mm/s, the average failure load results are consistent with milling the composite to a depth of 1 mm.

Two main conclusions can be drawn from this study. The first concerns the optimal feed rate of the welding heads, which, for the tested samples, was 0.25 mm/s. At this rate, the average failure loads of the welds showed much higher values than at the other tested feed rates: 0.19 mm/s and 0.31 mm/s. Another conclusion concerns the value of milling the composite reinforcing the profile. Based on the tests carried out, the optimum milling variant for the composite reinforcement cannot be clearly identified. At milling depths of 0.5 mm and 1 mm and with no milling of the composite, average failure loads of over 3450 N were obtained, but the differences between the results were not statistically significant. Therefore, the results of this study do not allow the identification of a single optimum milling depth.

## Figures and Tables

**Table 1:** Summary matrix of test variants, where D is the milling depth of the composite reinforcement [mm] and P is the feed rate of the welding heads [mm/s]. The 0.25 mm/s welding head speed variant was investigated previously in [25].

	D 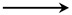		
**P** 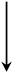	D = 0.0 P = 0.19	D = 0.5 P = 0.19	D = 1.0 P = 0.19
	D = 0.0 P = 0.25	D = 0.5 P = 0.25	D = 1.0 P = 0.25
	D = 0.0 P = 0.31	D = 0.5 P = 0.31	D = 1.0 P = 0.31

**Table 2:** Dimensional deviations of the welded molds for a set welding head speed of 0.31 mm/s when milling the composite reinforcement to a depth of 1 mm.

Sample No.	1	2	3
Dimensional deviations from nominal size in the X; Y axes [mm]	1; 1	1; 1	1; 1

**Table 3:** Dimensional deviations of the welded molds for a set welding head speed of 0.19 mm/s when milling the composite reinforcement to a depth of 1 mm.

Sample No.	1	2	3
Dimensional deviations from nominal size in the X; Y axes [mm]	1; 1	1; 1	1; 1

**Table 4:** Dimensional deviations of welded molds for a set welding head speed of 0.31 mm/s when milling the composite reinforcement to a depth of 0.5 mm.

Sample No.	1	2	3
Dimensional deviations from nominal size in the X; Y axes [mm]	1; 1	0; 1	1; 1

**Table 5:** Dimensional deviations of welded molds for a set welding head speed of 0.19 mm/s when milling the composite reinforcement to a depth of 0.5 mm.

Sample No.	1	2	3
Dimensional deviations from nominal size in the X; Y axes [mm]	0; 1	0; 1	0; 0

**Table 6:** Dimensional deviations of the welded molds for the set welding head speed of 0.31 mm/s without milling the composite reinforcement.

Sample No.	1	2	3
Dimensional deviations from nominal size in the X; Y axes [mm]	0; 0	0; 0	0; 0

**Table 7:** Dimensional deviations of welded molds for a set welding head speed of 0.19 mm/s without milling the composite reinforcement.

Sample No.	1	2	3
Dimensional deviations from nominal size in the X; Y axes [mm]	0; 1	0; 0	0; 1

## Data Availability

The original contributions presented in this study are included in the article. Further inquiries can be directed to the corresponding author.
